# C-955-06. Housing Temperature Alters the Pathophysiological and Proteomic Responses to Burn Injury in Mice

**DOI:** 10.1093/jbcr/irag033.155

**Published:** 2026-04-06

**Authors:** Meagan S Kingren, Lillie D Treas, Mary Barre, Abby Barlow, Jaycelyn Hall, Esther H Teo, Craig Porter

**Affiliations:** University of Arkansas for Medical Sciences, Little Rock, AR; Arkansas Children's Research Institute, Little Rock, AR; Arkansas Children's Research Institute, Little Rock, AR; Arkansas Children's Research Institute, Little Rock, AR; University of Arkansas for Medical Sciences, Little Rock, AR; University of Arkansas for Medical Sciences, Little Rock, AR; University of Arkansas for Medical Sciences, Little Rock, AR

## Abstract

**Introduction:**

While rodents are commonly used to study the hypermetabolic stress response to burns, most studies house mice at temperatures known to trigger a cold-induced hypermetabolic response and typically fail to consider sex as a biological variable. Here, we describe global proteomic responses to burns in male and female mice housed at standard (24°C) and thermoneutral (30°C) temperatures.

**Methods:**

C57BL/6 J mice (n = 64; 16 per group; 50% male) were housed at room temperature (24°C; RT) or thermoneutrality (30°C; TN) following sham or scald burn injury until euthanasia 14 days post injury. Burn injuries involved ~12.5% total body surface area (6.5 cm2 females; 8.6 cm2 males). Mice underwent metabolic phenotyping to assess energy expenditure (EE) post sham/burn, as well as pre-injury and euthanasia DEXA scans to determine body composition. Proteomic analyses were carried out on the liver, brown adipose tissue (BAT), white adipose tissue (WAT), quadriceps femoris (quad), heart, kidney, spleen, and plasma.

**Results:**

Housing mice below TN results in profound hypermetabolism, where resting energy expenditure (REE) is 70% greater in mice house at RT vs. TN. Burns resulted in moderate hypermetabolism in mice housed at TN only, evidenced by ~20% increase in REE (*p*<.05), suggesting that sub-thermoneutral housing masks burn induced hypermetabolism in mice. Cachexia was also more evident in mice housed at TN, where male mice subjected to burns had slower growth curves and demonstrated greater BAT and WAT wasting post burn when compared to RT (*p*<.05). At the molecular level, sub-TN housing masked the burn stress response. Notably, there were nearly 2-fold more differentially abundant proteins in burn injured mice housed at TN compared to RT. Acute phase response proteins were upregulated in several tissues in both sexes post burn, though this signal was more pronounced in female mice. Ingenuity pathway analysis and mitochondria-specific analysis using MitoCarta 3.0 related proteins revealed that changes in the mitochondrial proteome were much greater in TN housed mice compared to RT housed mice. In general, sexual dimorphisms in the global proteomic response to burns were more evident when mice were housed at TN.

**Conclusions:**

Sub-TN housing masks physiological and tissue specific proteomic responses to burn injury. Humanizing rodent physiology through TN housing enhances the utility of mice to model the stress response to burns.

**Applicability of Research to Practice:**

Extrinsic variables such as housing temperature must be controlled in order to enhance the rigor and reproducibility of rodent models of burns to increase their translational value.

**Funding for the study:**

NIGMS.

